# Supplementary material for: Social prescribing for children and young people in the UK: characterising access and care pathways using electronic health records
Source: BMJ Open. 2026 Sep 28;16(9):e120888. doi: 10.1136/bmjopen-2026-120888 (PMC13629868; doi:10.1136/bmjopen-2026-120888)
Supplement: Supplementary file 1 [file bmjopen-16-9-s001.pdf]

Supplementary Materials

Table S1. Number of children and young people and proportion of sample (n=52,585) with missing data.

Characteristic	Missing
Age	-
Gender	-
Ethnicity	43,555 (83%)
Country	-
Urbanicity	12,688 (24%)
Index of multiple deprivation (IMD)	12,688 (24%)
Referral source	5,516 (10%)
Referral reason	162 (<1%)
Case status	-
Number of contacts	-
Year referred	6,358 (12%)
Contact type[a]	22,191 (42%)
Contact length[a]	35,257 (67%)
Link worker time[a]	35,257 (67%)
Number of signposts	-
Number of interventions	-
Intervention format	-
Intervention domain[b]	2,104 (12%)

[a] Includes those with no contacts recorded (n=6,357) as well as those with contacts recorded but this information missing (n=15,834 for contact type; n=28,900 for contact length/link worker time).
[b] Percentage of individuals with one or more interventions recorded (n=18,094), i.e. for whom these data should not be missing.

Table S2. Characteristics of children and young people referred to social prescribing, shown for the full analytical sample and limited to individuals with two or more referrals.

	**Analytical sample** N=52,585	**Individuals with 2+ referrals** N=7,330
**Characteristic**	**Mean (SD)**	
Age (years)	20.04 (4.71)	21.00 (3.84)
	**Percent**	
Gender		
Female	57%	59%
Male	39%	36%
Non-binary	0.3%	0.3%
Transgender	0.4%	0.4%
Other	0.5%	1.0%
Not disclosed	3%	2.6%
Country		
England	79%	77%
Wales	11%	18%
Scotland	3%	2%
Northern Ireland	7%	3%
Residential area urban	91%	92%
In lowest IMD decile	28%	30%
Medical referral	79%	83%
Referral reason (multiple allowed)		
Mental health	44%	45%
Physical health	11%	8%
Social	16%	17%
Lifestyle	16%	13%
Employment/education	16%	16%
Practical	27%	36%
Other	12%	13%
Year referred		
2017	0.4%	0.7%
2018	2%	2%
2019	5%	5%
2020	6%	7%
2021	15%	18%
2022	21%	22%
2023	19%	20%
2024	18%	17%
2025	14%	8%

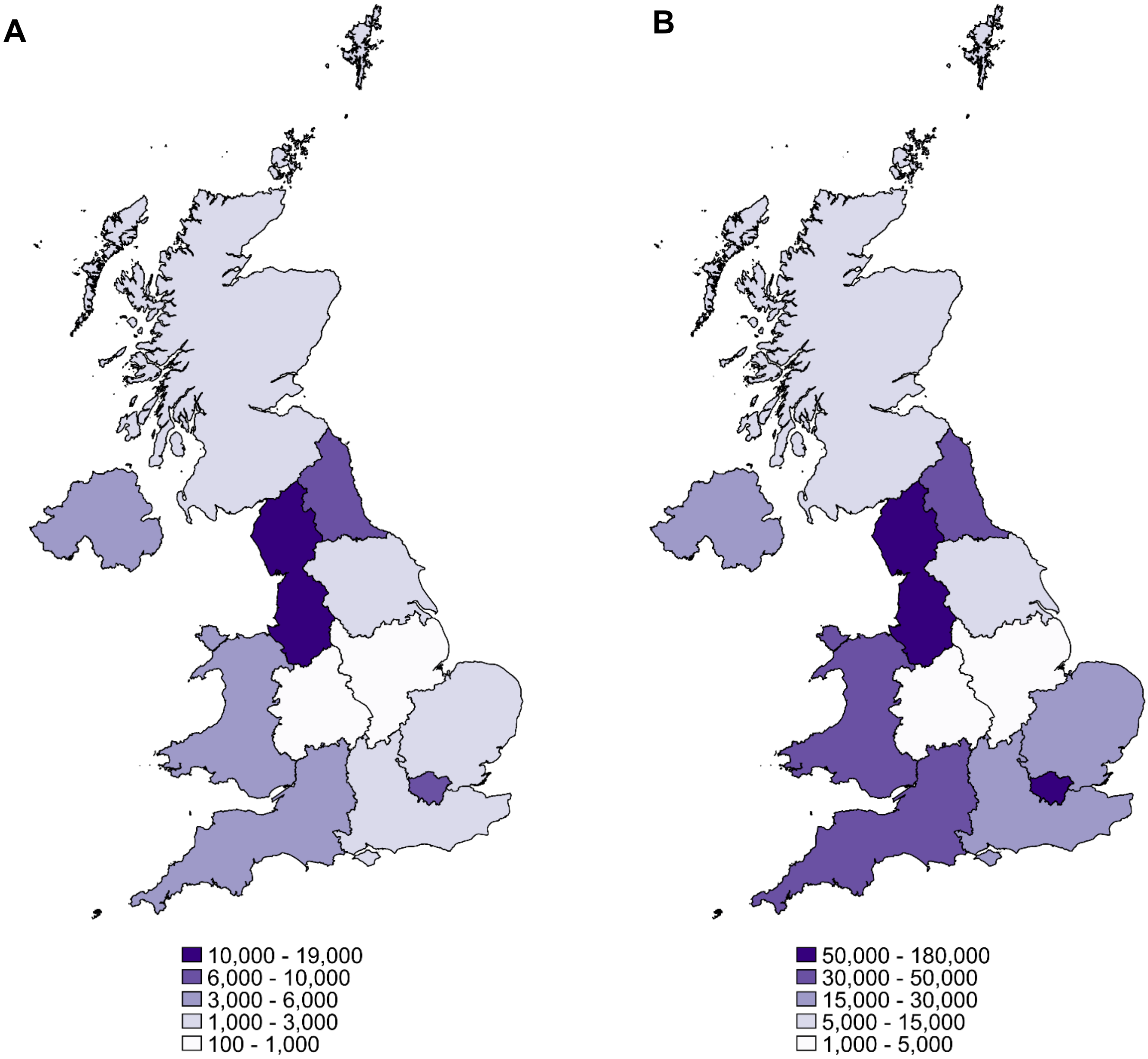

Figure S1. Maps showing A) the distribution of children and young people included in the analytical sample, compared to B) the distribution of all individuals (of any age) included in the Access Elemental dataset.

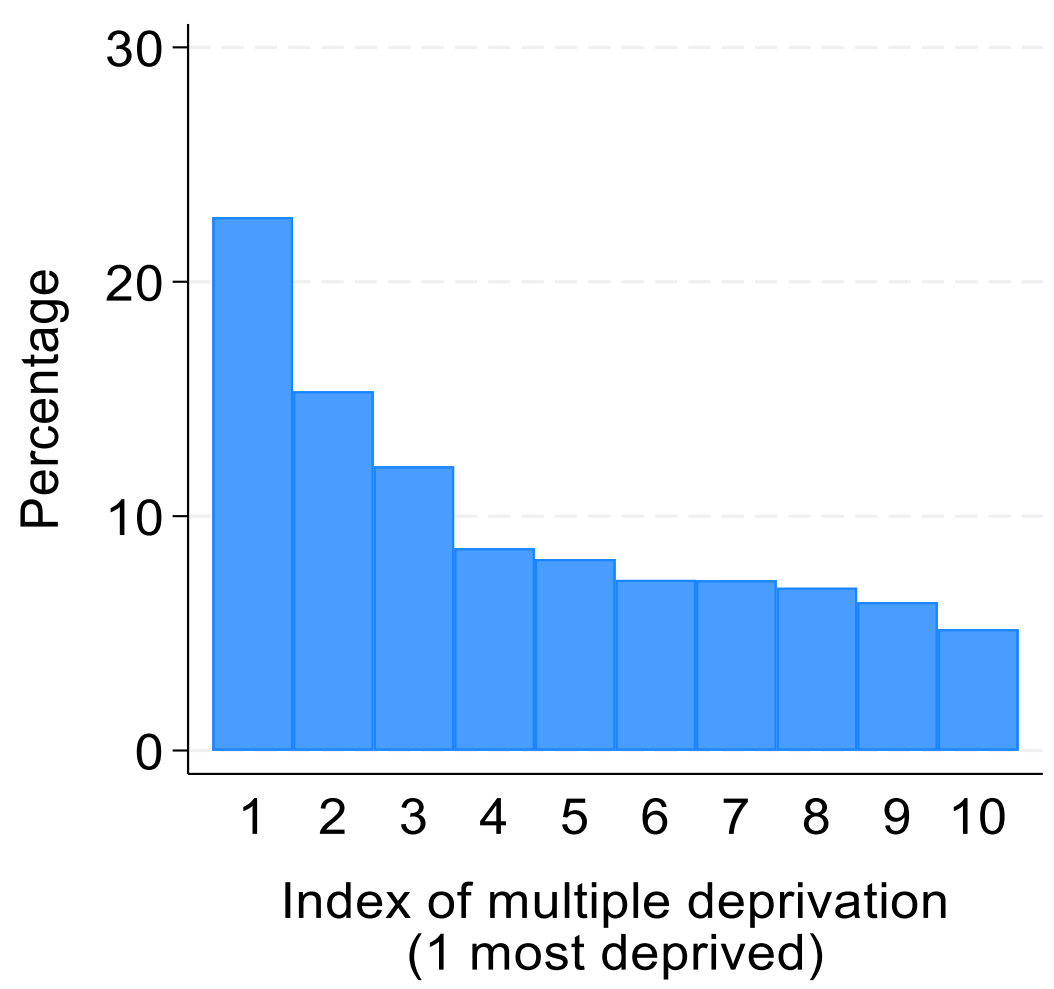

Figure S2. Descriptive statistics showing neighbourhood index of multiple deprivation for all individuals (of any age) included in the Access Elemental dataset (n=395,762).

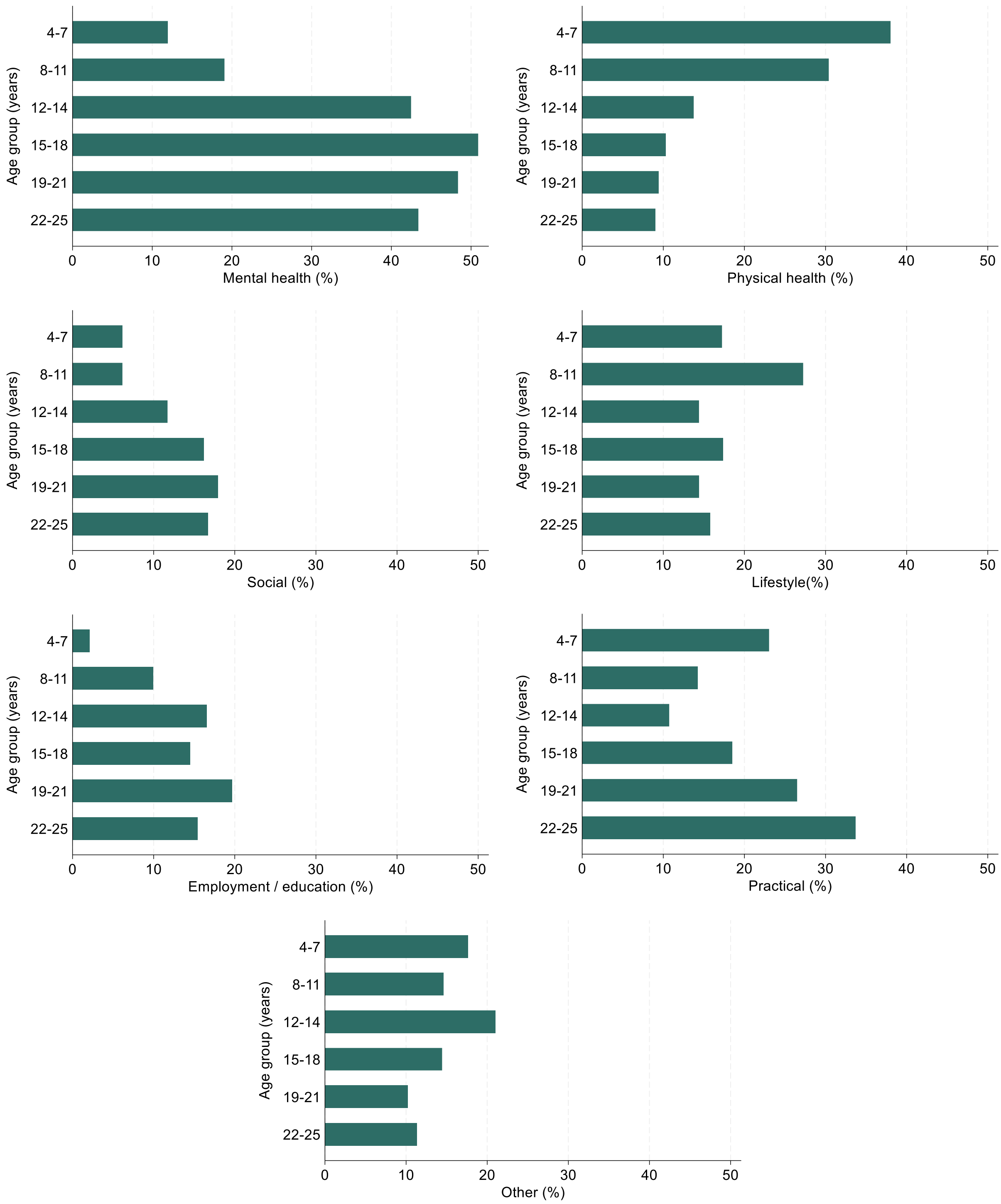

Figure S3. Descriptive statistics showing the difference in referral reasons according to age group (n=52,423). Each individual case may have multiple referral reasons.

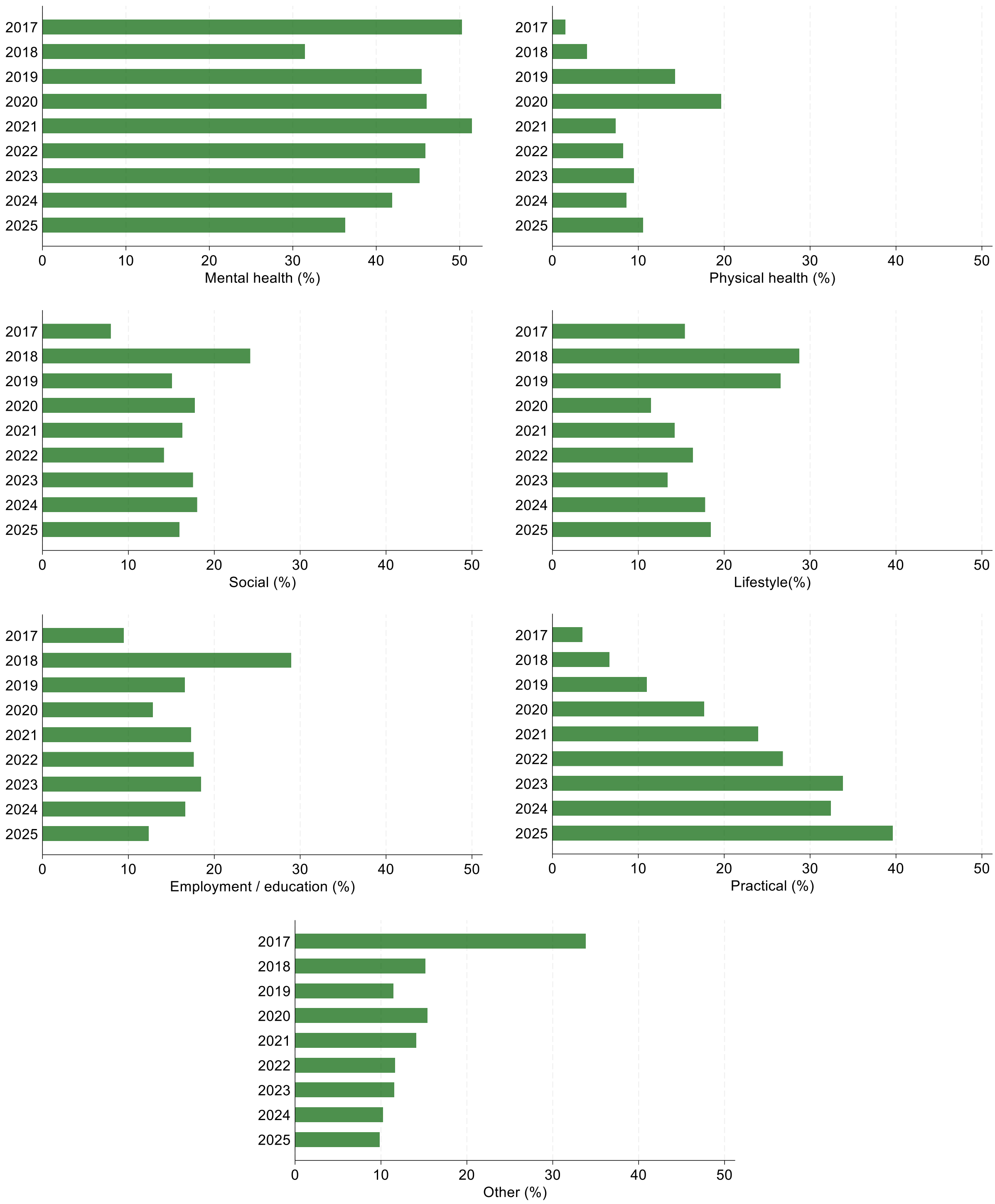

Figure S4. Descriptive statistics showing the difference in referral reasons according to the year in which individuals received social prescribing (n=46,068). Each individual case may have multiple referral reasons.

Sensitivity analyses: multiple imputation

Table S3. Characteristics of children and young people referred to social prescribing before and after multiple imputation for imputed variables (N=52,585; 40 imputed datasets).

	Raw data	Imputed
**Characteristic**	**Percent**	
Residential area urban	91%	90%
In lowest IMD decile	28%	28%
Medical referral	79%	77%
Referral reason (multiple allowed)		
Mental health	44%	44%
Physical health	11%	11%
Social	16%	16%
Lifestyle	16%	16%
Employment/education	16%	16%
Practical	27%	27%
Other	12%	12%
Year referred		
2017	0.4%	0.4%
2018	2%	2%
2019	5%	5%
2020	6%	6%
2021	15%	15%
2022	21%	21%
2023	19%	19%
2024	18%	18%
2025	14%	14%

Table S4. Results from regression models testing the associations between age (continuous years) and individual characteristics in 40 imputed datasets.

	Model type	N	Coef	95% CI	p value
**Gender** (ref: female)	Multinomial logistic	52,585			
Male	(RRR)		**0.96**	**0.96, 0.97**	**<0.001**
Non-binary			1.02	0.98, 1.05	0.307
Transgender			1.01	0.98, 1.04	0.444
Other			1.03	0.99, 1.07	0.090
Not disclosed			1.01	0.98, 1.05	0.501
**Medical referral source**	Logistic (OR)	52,585	**1.08**	**1.00, 1.17**	**0.045**
**Referral reason**					
Mental health	Logistic (OR)	52,585	**1.04**	**1.00, 1.09**	**0.032**
Physical health/wellbeing	Logistic (OR)	52,585	**0.91**	**0.83, 0.99**	**0.031**
Social relationships	Logistic (OR)	52,585	**1.05**	**1.02, 1.07**	**0.001**
Lifestyle	Logistic (OR)	52,585	0.98	0.91, 1.05	0.584
Employ/education/skills	Logistic (OR)	52,585	1.03	0.99, 1.07	0.105
Practical support	Logistic (OR)	52,585	**1.08**	**1.03, 1.14**	**0.002**
Other reasons	Logistic (OR)	52,585	0.96	0.92, 1.01	0.085

Note. Bold text indicates p<0.05.

Table S5. Results from regression models testing the associations between year in which social prescribing was received and individual characteristics in 40 imputed datasets.

	Model type	N	Coef	95% CI	p value

**Age**	Linear (B)	52,585	**0.30**	**0.06, 0.55**	**0.014**
**Gender** (ref: female)	Multinomial logistic (RRR)	52,585			
Male			1.02	1.00, 1.05	0.055
Non-binary			**1.22**	**1.10, 1.36**	**<0.001**
Transgender			**1.17**	**1.06, 1.29**	**0.001**
Other			1.04	0.82, 1.32	0.742
Not disclosed			**1.32**	**1.01, 1.71**	**0.040**
**Medical referral source**	Logistic (OR)	52,585	1.08	0.93, 1.25	0.312
**Referral reason**					
Mental health	Logistic (OR)	52,585	0.95	0.88, 1.03	0.219
Physical health/wellbeing	Logistic (OR)	52,585	0.95	0.87, 1.04	0.272
Social relationships	Logistic (OR)	52,585	1.00	0.94, 1.07	0.948
Lifestyle	Logistic (OR)	52,585	0.97	0.85, 1.12	0.712
Employ/education/skills	Logistic (OR)	52,585	0.96	0.89, 1.03	0.224
Practical support	Logistic (OR)	52,585	**1.24**	**1.13, 1.37**	**<0.001**
Other reasons	Logistic (OR)	52,585	**0.92**	**0.86, 0.98**	**0.005**

Note. Bold text indicates $p<0.05$.
