# Supplementary material for: Social prescribing for children and young people in the UK: characterising access and care pathways using electronic health records
Source: BMJ Open. 2026 Sep 28;16(9):e120888. doi: 10.1136/bmjopen-2026-120888 (PMC13629868; doi:10.1136/bmjopen-2026-120888)
Supplement: Supplementary file 2 [file bmjopen-2026-120888.reviewer_comments.pdf]

PEER REVIEW HISTORY

BMJ Open publishes all reviews undertaken for accepted manuscripts. Reviewers are asked to complete a checklist review form and are provided with free text boxes to elaborate on their assessment. These free text comments are reproduced below.

ARTICLE DETAILS

Title (Provisional)

Social prescribing for children and young people in the UK: characterising access and care pathways using electronic health records

Authors

Bone, Jessica K; Bu, Feifei; Hayes, Daniel; Fancourt, Daisy

VERSION 1 - REVIEW

**Reviewer**	**1**
**Name**	**Gangadharan, Satheesh**
**Affiliation**	**Loughborough University Loughborough School of Design and Creative Arts, Design school**
**Date**	**15-Jun-2026**
**COI**	**None**

This is a good paper that makes an important contribution by examining social prescribing among children and young people using a large national dataset. The study is clearly written, and highly relevant to current UK policy discussions, particularly around health inequalities and community-based care. Its main strengths lie in the large sample size, the focus on younger populations, and the use of real-world data to describe how services are actually delivered in practice. Overall, it is suitable for publication, but some further changes as below.

1. selection bias. The analysis is based entirely on data from services that use the Access Elemental platform, and while this is acknowledged, the implications are not fully explored. It remains unclear how representative these services are of social prescribing provision across the UK, and some findings—such as the geographic distribution of participants—may reflect platform uptake rather than genuine variation in access. This limitation should be emphasised more strongly, particularly when drawing system-level conclusions.

2. Missing data is another important concern. Very high levels of missingness are reported for key variables, including ethnicity and measures of contact time. While the authors note data quality issues, the potential impact of missing data on estimates and conclusions is not

fully examined. A more detailed discussion of how this might influence results would strengthen the paper.

3. Some interpretations, particularly around access and inequalities, could be made more cautious. For example, the conclusion that social prescribing has “limited reach” for those under 18 is plausible, but the data only include individuals who have already been referred. Without information on the wider population who could benefit from social prescribing, it is not possible to quantify access or unmet need directly.

4. There is also some ambiguity around what the outcomes represent. Measures such as contacts, signposts, and interventions are based on recorded activity rather than confirmed service delivery or engagement. The fact that only a minority of participants have recorded interventions raises important questions, but it is unclear whether this reflects limited provision or incomplete recording (particularly with a messy data).

5. From a methodological perspective, the use of unadjusted regression models limits the extent to which associations can be interpreted as independent effects. While this approach may be appropriate for descriptive purposes, it would be helpful to justify this choice more explicitly and to clarify that the results should not be interpreted causally.

Despite these issues, the study has several notable strengths. It provides the first large-scale overview of social prescribing pathways for children and young people in the UK, offers valuable insight into referral reasons and types of support provided, and highlights important gaps in service delivery—particularly the relatively low proportion of individuals receiving recorded interventions.

**Reviewer**	**2**
**Name**	**Kelson, Zoe**
**Affiliation**	**University of Exeter, Mathematics**
**Date**	**04-Aug-2026**
**COI**	**None**

This study aims to describe the characteristics of children and young people referred to social prescribing across the UK and understand what social prescribing looks like for these young people.

Reviewer comments:

Can statistical modelling methods and outcomes please also be summarised in the Abstract?

"we aimed to explore whether access to and experiences of social prescribing vary with age and have changed from 2017 to 2025" [Abstract]

Can the authors please consider the impact of COVID-19 on the data included in the study?

"Social prescribing hubs and services across the UK that use Access Elemental (a cloud-based social prescribing platform)...

52,585 individuals referred to social prescribing in 2017-2025 aged 4-25 years (mean=20.04, SD=4.71), of whom 57% were female, 39% male, <2% were in other gender groups, and 3% did not disclose their gender" [Abstract]

and

"Access Elemental is a cloud-based SP platform used by health and social care professionals, community development workers, and other service providers to keep track of SP activities and their impact from the point of referral (https://www.theaccessgroup.com/en-gb/our-brands/elemental/). Access Elemental data are available since January 2017, and this study used data up to 30th August 2025...

This left a final analytical sample size of 52,585 CYP, who were from over 700 SP sites across all regions of the UK."

Can the authors please further comment on whether the included cohort can be considered to be representative?

Can the cohort characteristics and demographics please be tabulated?

"For CYP with more than one case of SP recorded, only the first case was retained (15% cases excluded). "

Can the characteristics of CYP with more than one case of SP also be explored by the authors?

"Reporting follows the RECORD guidelines."

Thanks to the authors for providing a copy of the associated checklist.

"Access Elemental records basic demographic information, including age at time of referral (years), gender, and ethnicity. It categorised gender as female, male, non-binary, transgender, other, or not disclosed (not yet disclosed, prefer not to say). Ethnicity could not be used due to the high proportion of missing data (83% missing; Table S1), as in previous analyses of Access Elemental and other SP records.

We determined country of residence (England, Wales, Scotland, Northern Ireland) from individuals' postcodes. Where postcodes were missing (24%), we used the SP site location...

We could not examine the domain or format of signposts because this information was missing for 77% of individuals. "

Can the authors please assess whether missing data can be be assumed to be missing at random?

"Over 600 reasons for referral were recorded, which we grouped into seven domains. Grouping was done independently by two authors with disagreements discussed and resolved by consensus, in line with previous analyses of Access Elemental data"

Can the authors please assess inter-rater agreement prior to consensus?

"evaluated whether all individual characteristics and the SP pathway differed according to age, using a series of unadjusted regression models to test their associations with age (measured continuously in years). We used logistic regression for binary outcomes, multinomial logistic regression for categorical outcomes, and negative binomial regression for count outcomes (due to overdispersion). All regression models included clusterrobust standard errors to account for clustering of individuals within SP sites. To plot differences according to age, we classified participants into age groups following the key stages of UK educational provision: 4-7, 8-11, 12-14, 15-18, 19-21, and 22-25 years. Finally, to explore how the characteristics of CYP referred to SP have changed over time, we used separate regression models to test the associations between the year in which SP was delivered and all individual characteristics, specifically age (linear regression), gender (multinomial logistic regression), referral source (logistic regression), and referral reasons (logistic regression)"

Appropriate modelling methods have been applied by the authors.

Can the authors please consider applying formal trend analyses, such as joinpoint regression modelling to assess changes over time?

"Missing data were handled using listwise deletion separately for each analysis due to the lack of auxiliary variables to enable multiple imputation"

and

"Table S1. Number of children and young people and proportion of sample (n=52,585) with missing data."

Can a sensitivity analysis on the approach taken for handling missing data please be performed?

Can the Figures please also show statistical outcomes?

"Administrative data were limited by missingness and varying data quality, meaning we could not explore the role of ethnicity among other factors • It was unclear whether individuals with no recorded interventions did not receive any prescriptions or were missing these data • We could only explore access to and delivery of social prescribing by sites that have chosen to use the Access Elemental platform, making it difficult to understand what was driving changes over time" [Strengths and Limitations of this study]

and

"However, administrative data are limited by missingness and varying data quality. For example, we could not explore the role of ethnicity, as it was missing for the large majority of CYP. It was unclear whether individuals with no recorded signposts/interventions did not

receive any prescriptions or were missing these data. Additionally, we could not investigate referral source in further detail than medical vs non-medical routes, which remains a key priority for CYP SP. Enhancing recording and data quality thus remain key priorities for SP practitioners and platform providers, as previously identified.7,14,36,48 Furthermore, due to the nature of our data, we could only explore access to and delivery of SP by sites that have chosen to use the Access Elemental platform. We could not test whether residential characteristics of referred CYP or SP pathways differed over time, as these were considered likely to result from differences in the SP sites who chose (or could afford) to use Access Elemental over time. Relatively few SP sites were consistently included in this dataset from 2017 to 2025. It was also unclear whether evidence for changes in CYP characteristics over time (e.g. increasing age) were driven by these changes in SP sites. Testing how CYP SP has changed during this period, independent of the platform used to deliver SP, should be a focus of future research. Nevertheless, our findings were generally in line with a previous evaluation of CYP SP that used data from the Joy platform,36 indicating that they were not solely driven by the use of data from Access Elemental."

The authors have provided a Discussion on the study limitations.

VERSION 1 - AUTHOR RESPONSE

Reviewer 1

Prof. Satheesh Gangadharan, Loughborough University Loughborough School of Design and Creative Arts, Leicestershire Partnership NHS Trust

This is a good paper that makes an important contribution by examining social prescribing among children and young people using a large national dataset. The study is clearly written, and highly relevant to current UK policy discussions, particularly around health inequalities and community-based care. Its main strengths lie in the large sample size, the focus on younger populations, and the use of real-world data to describe how services are actually delivered in practice. Overall, it is suitable for publication, but some further changes as below.

Reply: Thank you for your kind comments and useful feedback, which we have addressed point by point below.

1. Selection bias. The analysis is based entirely on data from services that use the Access Elemental platform, and while this is acknowledged, the implications are not fully explored. It remains unclear how representative these services are of social prescribing provision across the UK, and some findings—such as the geographic distribution of participants—may reflect platform uptake rather than genuine variation in access. This limitation should be emphasised more strongly, particularly when drawing system-level conclusions.

Reply: We agree that this is an important limitation of our study. We have further developed the implications of relying on data from Access Elemental when we first discuss the geographic

distribution of participants in the second paragraph of the discussion (page 10). We have also further built on this in the limitations section (page 12).

2. Missing data is another important concern. Very high levels of missingness are reported for key variables, including ethnicity and measures of contact time. While the authors note data quality issues, the potential impact of missing data on estimates and conclusions is not fully examined. A more detailed discussion of how this might influence results would strengthen the paper.

Reply: Again, we agree that this is a key issue, and we hope to make this clear throughout the manuscript. We have added a sentence highlighting that "analyses were limited by missingness and varying data quality throughout" to the first paragraph of the discussion (page 9). In the limitations section, we have further developed the discussion of the potential impact of missing data (page 12). We consider several potential sources of bias and the implications for our findings. We also hope that the addition of sensitivity analyses using multiple imputation, as recommended by reviewer 2, strengthens our paper. Additionally, considering the challenges with missingness alongside points 3-4 below, we have added more caveats to our interpretation of the findings throughout the discussion, including emphasizing that number of contacts/interventions rely on recorded information.

3. Some interpretations, particularly around access and inequalities, could be made more cautious. For example, the conclusion that social prescribing has "limited reach" for those under 18 is plausible, but the data only include individuals who have already been referred. Without information on the wider population who could benefit from social prescribing, it is not possible to quantify access or unmet need directly.

Reply: Thank you for noting this, as we do not want to overstate our findings. We have toned down our interpretation throughout the discussion, particularly where we focus on access and inequalities (pages 9-12) and the conclusion (page 12).

4. There is also some ambiguity around what the outcomes represent. Measures such as contacts, signposts, and interventions are based on recorded activity rather than confirmed service delivery or engagement. The fact that only a minority of participants have recorded interventions raises important questions, but it is unclear whether this reflects limited provision or incomplete recording (particularly with a messy data).

Reply: This is another key limitation, which we hope our edits have highlighted more clearly. As noted above, we have now made sure we talk about "recorded" contacts/ interventions/ signposts throughout. We explicitly state that these might underestimate actual figures when the intervention measure is first described (page 6). We also explore this in more detail in the discussion (page 11).

5. From a methodological perspective, the use of unadjusted regression models limits the extent to which associations can be interpreted as independent effects. While this approach may be appropriate for descriptive purposes, it would be helpful to justify this choice more

explicitly and to clarify that the results should not be interpreted causally.

Reply: We have now clarified in the statistical analysis section that this was a descriptive analysis. Given that the causal pathways are likely interrelated, but the directions of effects unclear, each outcome was tested in a separate unadjusted model. We also explicitly state that "These models only aimed to describe differences over time, so results should not be interpreted causally" (page 6).

Despite these issues, the study has several notable strengths. It provides the first large-scale overview of social prescribing pathways for children and young people in the UK, offers valuable insight into referral reasons and types of support provided, and highlights important gaps in service delivery—particularly the relatively low proportion of individuals receiving recorded interventions.

Reply: Thank you again for highlighting these strengths.

Reviewer 2

Prof. Zoe Kelson, University of Exeter

This study aims to describe the characteristics of children and young people referred to social prescribing across the UK and understand what social prescribing looks like for these young people.

Can statistical modelling methods and outcomes please also be summarised in the Abstract? *"we aimed to explore whether access to and experiences of social prescribing vary with age and have changed from 2017 to 2025"* [Abstract]

Reply: We have added a description of the modelling methods in the Primary and secondary outcome measures section of the abstract "We summarised young people's characteristics (age, gender, country, urban area, area deprivation, referral route, reason for referral) and the care pathway received (case status, number type and length of contacts, onward signposting and interventions) using descriptive statistics. We then used unadjusted linear, logistic, multinomial logistic, and negative binomial regression models to describe whether these factors differed by age and over time" (page 2).

Can the authors please consider the impact of COVID-19 on the data included in the study?

Reply: We have added discussion of the impact of Covid-19 on our data to the limitations section of the discussion: "However, the study period did include the Covid-19 pandemic, which is likely to have influenced SP pathways, as appointments may have moved online and access to in-person interventions was likely reduced. We did not observe a drop in the number of CYP referred to SP during 2020, but referral rates did increase dramatically from 2021 onwards. It is not clear whether this was driven by post-pandemic changes or the national

rollout of funding for link workers. Future research should thus explore whether SP needs to be adapted for use during public health emergencies” (page 12).

"Social prescribing hubs and services across the UK that use Access Elemental (a cloud-based social prescribing platform)... 52,585 individuals referred to social prescribing in 2017-2025 aged 4-25 years (mean=20.04, SD=4.71), of whom 57% were female, 39% male, <2% were in other gender groups, and 3% did not disclose their gender" [Abstract]
and
"Access Elemental is a cloud-based SP platform used by health and social care professionals, community development workers, and other service providers to keep track of SP activities and their impact from the point of referral (https://www.theaccessgroup.com/en-gb/our-brands/elemental/). Access Elemental data are available since January 2017, and this study used data up to 30th August 2025...
This left a final analytical sample size of 52,585 CYP, who were from over 700 SP sites across all regions of the UK."
Can the authors please further comment on whether the included cohort can be considered to be representative?

Reply: Unfortunately, it is difficult to assess whether this cohort is representative, as there are no other population-level statistics on the CYP referred to SP in the UK. We would not expect these individuals to be a representative sample of all CYP in the UK, as not everyone needs referral to SP. The best we can do is compare the distribution across the UK of this cohort of CYP to the distribution of all individuals in Access Elemental. This is illustrated in Figure S1 and described in the descriptive statistics (page 7), as well as in the discussion (page 10).

Can the cohort characteristics and demographics please be tabulated?

Reply: The demographic and clinical characteristics are tabulated in Table S2. We have not included this in the main body of the manuscript because it would duplicate information included in Figures 1-2 and in the text.

"For CYP with more than one case of SP recorded, only the first case was retained (15% cases excluded)."
Can the characteristics of CYP with more than one case of SP also be explored by the authors?

Reply: We have added a column showing the characteristics of CYP with more than one case of SP to Table S2. In the data section of the main text, we now reference this table and note that these individuals had very similar characteristics to those with only one case of SP (page 4).

"Reporting follows the RECORD guidelines."
Thanks to the authors for providing a copy of the associated checklist.

Reply: Thanks, we are glad you found this helpful.

Can the authors please assess whether missing data can be assumed to be missing at random?

Reply: Based on our previous work with Elemental data, it is fair to assume that missingness in the individual characteristics (urbanicity, IMD, referral source, referral reason, year referred) is strongly related to other observed variables. Exploratory analyses confirmed this, as missingness was associated with fully observed variables age, gender, country, case status, and number of contacts. This suggests that missing at random is a plausible assumption. We have not analysed variables with extremely high proportions missing (>75%; ethnicity, domain/format of signposts), as we do not think the missing at random assumption will hold.

"Over 600 reasons for referral were recorded, which we grouped into seven domains. Grouping was done independently by two authors with disagreements discussed and resolved by consensus, in line with previous analyses of Access Elemental data"
Can the authors please assess inter-rater agreement prior to consensus?

Reply: We have now specified that "inter-rater agreement before consensus was 78%" for the referral reasons (page 5) and was 74% for the intervention domains (page 6). This is acceptable given the exploratory nature of these analyses and the conceptual overlap between reasons/domains.

Appropriate modelling methods have been applied by the authors.

Reply: Thank you.

Can the authors please consider applying formal trend analyses, such as joinpoint regression modelling to assess changes over time?

Reply: As noted in the limitations, we could only explore access to and delivery of SP by sites that chose to use the Access Elemental platform, and few sites were consistently in the dataset for the whole study period. Changes in CYP characteristics over time may therefore be driven by these changes in SP sites, as opposed to actual changes in practice. We are therefore hesitant to include a formal trend analysis, such as joinpoint regression, as we do not want to make definitive conclusions about the time trends in CYP SP. The dataset also only includes 9 years of data, which is a relatively low number of timepoints for an exploratory joinpoint regression. This is particularly problematic when combined with the small sample sizes in the first few years, even before missing data on individual characteristics are accounted for (2017 n=201, 2018 n=922, 2019 n=2246, vs 2022 n=9854). We realise the imbalanced sample size across years was only briefly mentioned in the manuscript previously, so we have added this information to the descriptive statistics table (Table S2). We also highlight that testing how CYP SP has changed during this period, independent of the platform used to deliver SP, should be a focus of future research (page 11).

Following the reviewer's suggestion, we tried using exploratory piecewise regression models to test for non-linear trends in CYP characteristics over time. However, we found no evidence for

any breakpoints, indicating that either there were no non-linearities or our analyses were insufficiently powered to detect them.

"Missing data were handled using listwise deletion separately for each analysis due to the lack of auxiliary variables to enable multiple imputation" and *"Table S1. Number of children and young people and proportion of sample (n=52,585) with missing data."*
Can a sensitivity analysis on the approach taken for handling missing data please be performed?

Reply: We have now added a sensitivity analysis using multiple imputation with chained equations (MICE) to impute missing data on individual characteristics (urbanicity, IMD, referral source, referral reason, year referred). We used logistic and ordered logistic regression and predictive mean matching (with 5 nearest neighbours) according to variable type, generating 40 imputed datasets (39% of individuals had missing data on one or more of these variables). The imputation model included all individual characteristics and variables describing the SP pathway with no missing values (Table S1). We have added findings using the 40 imputed datasets to the supplement (Tables S3-S5) and described them in a new sensitivity analyses section the main text (page 9). Findings did not change substantially, but we have highlighted two small differences following MICE. Overall, these sensitivity analyses suggest that our findings were robust to the approach for handling missing data.

There was also missingness in variables describing the SP pathway. However, it was not appropriate to impute these variables, as it is unclear whether missing values should be replaced. Missing values in contact type, contact length, and link worker time includes those with no contacts recorded as well as those with contacts recorded and these variables missing. Where no contact was recorded, it is not clear whether this was a true zero or whether it represented missing data, leaving us uncertain about which missing values should be imputed. We have the same concerns around imputing information on intervention domain.

Can the Figures please also show statistical outcomes?

Reply: We are concerned that adding statistical outcomes will make the figures difficult to read, particularly as they already include a lot of information, with many sub-panels per figure. Additionally, the figures all show descriptive statistics (not model estimates), so we want to avoid misinterpretation of statistical outcomes by adding them to descriptive plots. We have also found in previous work that adding statistical outcomes, such as asterisks to indicate significance, can encourage readers to interpret results solely based on the arbitrary p=0.05 threshold (which we hope to avoid).

The authors have provided a Discussion on the study limitations.

Reply: Thank you. As outlined above, we have also developed the discussion further based on both reviewers' comments.

VERSION 2 - REVIEW

Reviewer 1

Name Gangadharan, Satheesh

Affiliation Loughborough University Loughborough School of Design and Creative Arts, Design school

Date 08-Sep-2026

COI

The revised manuscript is noticeably stronger, particularly regarding missing data, platform-related selection bias, and the interpretation of recorded interventions. Although not completely, authors have addressed the issues raised in the previous review as best as possible. I am happy to recommend this paper for consideration by the editor for publication.
